# Exposome approach for identifying modifiable factors for the prevention of colorectal cancer

**DOI:** 10.1038/s41598-022-25832-9

**Published:** 2022-12-14

**Authors:** Nanqian Chen, Hailun Liang, Tao Huang, Ninghao Huang

**Affiliations:** 1 School of Public Administration and Policy, Renmin University of China, 59 Zhongguancun, Beijing, 100191 China; 2 Department of Epidemiology and Biostatistics, School of Public Health, Peking University, Beijing, China; 3 Department of Global Health, School of Public Health, Peking University, Beijing, China; 4 Key Laboratory of Molecular Cardiovascular Sciences (Peking University), Ministry of Education, Beijing, China; 5 Center for Intelligent Public Health, Institute for Artificial Intelligence, Peking University, Beijing, China

**Keywords:** Cancer prevention, Environmental impact, Risk factors

## Abstract

Previous studies have shown certain exposure factors (such as lifestyle and metabolism) are associated with colorectal cancer (CRC) events. However, the application of the exposome theoretical frame and the extent to which the exposome domain can modulate the risk of CRC remain unknown. Our study aimed to construct valid exposome measurements and examine the relationship between exposome counts and the risk of CRC. This study included 335,370 individuals in the UK Biobank. We used exploratory factor analysis to identify a valid construct of exposome factors. We then summed the exposome counts within each domain. Cox proportional hazard models were used to estimate the hazard ratios and 95% confidence intervals of CRC risk related to the exposome factors and counts. During an 8.69 year median follow-up, 10,702 CRC cases were identified. Five domains were extracted from 12 variables, including ecosystem, lifestyle, tobacco and alcohol use, social economics, and social support. The Cox model results showed that the ecosystem was positively related to the reduced CRC risk (HR = 0.970; 95% CI 0.952–0.989). Similar results were also found among the domains of healthy lifestyles (HR = 0. 889; 95% CI 0.871–0.907), and no tobacco and alcohol use (HR = 0.892; 95% CI 0.876–0.909). The disadvantageous social economic (HR = 1.081; 95% CI 1.058–1.105) and insufficient social support domains (HR = 1.036; 95% CI 1.017–1.056) were associated with an increased risk of CRC. Similar risk trends were also observed across the exposome count groups with CRC incidence. Our findings suggest that certain exposure domains are related to the incidence of CRC. Ecosystem, lifestyle, and social factors can be incorporated into prediction models to identify individuals at high risk of CRC.

## Introduction

Colorectal cancer (CRC) is the third most commonly diagnosed malignancy and the second leading cause of cancer-related deaths worldwide^1^. In the UK, more than 42,000 new diagnosed cases are reported^2^. In addition, colorectal cancer is the 4th most common cancer in the UK, accounting for 11% of all new annual cancer cases^2^. The lack of apparent symptoms in the early stages of CRC causes a heavy disease burden for people^3^. The majority of patients are diagnosed at the late stage, leading to a poor five-year net survival of 10% in the UK^2,4^.

CRC is a multifactorial disease with pathogenesis related to internal and external factors. Common internal risk factors include inflammation, dynamics of gut microbiota^5,6^, genetics^7^, age^8^, sex^9^, and race/ethnicity^9^. Various external exposures such as diet, smoking, drinking^10–13^, cancer screening^14^, socioeconomic status (SES,^15,16^, social support^17^, industrial pollution^18,19^, and other factors are associated with CRC events.

Environmental factors have been increasingly recognised to play an important role in diseases^20,21^. Resultingly, a new approach is necessary to elucidate carcinogenesis to inform early detection strategies to modulate the risks of CRC. The exposome approach aims to capture the diversity and range of complete environmental exposures in epidemiological studies, providing a comprehensive description of various exposures^21^. However, the influence of all exposome factors on health outcomes is poorly understood^22^. This means that few studies explored the whole set of exposome factors from a macro perspective to reveal the whole mechanism of modifiable factors in CRC development. To bridge this knowledge gap, we used UK Biobank (UKB) data to explore the relationship between exposome factors and CRC.

## Theoretical framework and literature review

### Theoretical framework

The exposome approach was first promoted by Dr Christopher Paul Wild. The exposome approach refers to the lifelong environmental (non-genetic) exposure that may impact an individual during their lifespan^23^. Three broad categories of non-genetic exposures have been defined: internal, specific external, and general external^23^. However, how to incorporate the hierarchical structure into a putative exposome analysis is one complexity of the exposome framework. This complexity remains to be addressed^24^.

**Figure 1:**
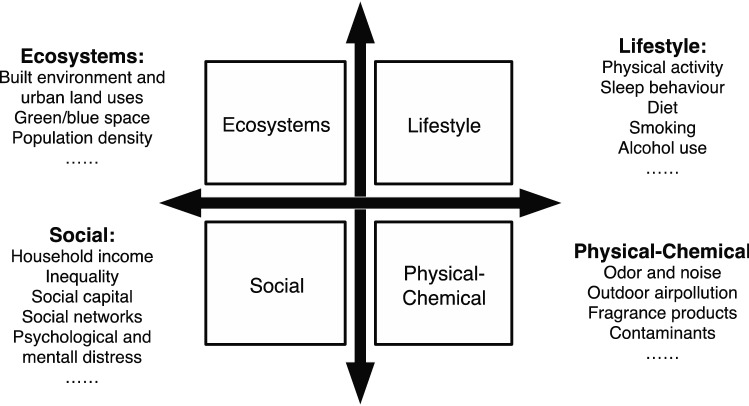
Exposome theoretical framework.

Based on Wild’s research, Vermeulen et al.^20^ developed an exposome model (see Fig. 1). They categorised the exposures into four parts: ecosystem, and lifestyle, social, and physical–chemical domains. The ecosystem domain includes food outlets, alcohol outlets, built environment, and green/blue space. The lifestyle domain includes physical activity, diet, and smoking. The social domain includes income, social networks, and psychological and mental stress. The physical–chemical domain includes air pollution, odour and noise, pesticides, water, and contaminants^20^. Compared with the original model, the Vermeulen model mapped a more detailed approach to utilising the exposome concept.

### Exposome factors and CRC

Previous studies have found that certain exposure factors are associated with CRC events. In this section, we divide the literature review into four parts to demonstrate the relationship between exposome factors and CRC.

First, in terms of lifestyle domain, numerous studies have suggested lifestyle factors to be associated with the risk of CRC^10–13^. A case–control study in Germany derived five modifiable lifestyle factors, namely smoking, alcohol consumption, diet, physical activity, and body fat^10^. This study found that adherence to a healthy lifestyle was associated with a reduced risk of CRC. A study using the UKB cohort also indicated that a healthier lifestyle (including body mass index [BMI], waist-hip ratio, physical activity, sedentary time, lower processed and red meat consumption, higher vegetable and fruit intake, lower alcohol consumption, and reduced tobacco smoking) contributed to a reduced risk of CRC^12^. A similar trend can also be seen in a study by Wang et al.^25^. The study used data from the Nurses’ Health Study (1988–2014) and the Health Professionals Follow-up Study (1988–2014), and suggested that a healthy lifestyle was associated with a lower incidence of CRC.

Second, in terms of social domain, few studies have focused on the influence of social determinants on the risk of CRC. Hastert et al. used data from the Vitamins and Lifestyle Study to examine the relationship between socioeconomic status and CRC incidence^26^. Living in the lowest SES areas was associated with a higher CRC incidence than those living in the highest SES areas. One study also indicated that a disadvantaged socioeconomic position in childhood was related to an increased risk of CRC^27^. Social support was another social factor that affected CRC events, however, the findings of the studies differed. One study in Copenhagen, Denmark found no significant association between social networks and CRC incidence^28^, while Ikeda et al. found that lower social support was associated with a higher incidence of CRC among men^29^.

Third, in terms of the ecosystem domain, few studies have evaluated the effect of ecosystems on CRC incidence. However, increasing studies have suggested that ecosystems play a role in cancer incidence^30,31^. A study from the United States suggested that higher neighbourhood walkability was correlated with lower incidence of multiple myeloma incidence^32^. Green space is a significant factor in cancer research. It has been regarded as a protective factor against mouth and throat^30^, skin^33^ and breast cancers^34^.

Fourth, in terms of the physical–chemical domain, pollution was significantly correlated with CRC incidence. Polychlorinated biphenyls and dioxins lead to food contamination and may increase the risk of bowel tumours^35^. An increased risk of CRC was also associated with living near industries. Pollutants such as nonylphenol, antimony, naphthalene, organotin compounds, and manganese increased the risk of CRC^18^. A study based on Canadian population found that exposure to chlorination by-products in public water supplies was associated with an increased risk of CRC in men^36^.

### The application of exposome framework in cancer

The application of the exposome framework can be seen in cancer studies^21,37,38^ and other non-communicable diseases, such as acne^39^, cardiovascular diseases^40^^,^^41^, and respiratory diseases^42^. Juarez et al.^43^ analysed lung cancer mortality disparities. They used an exposome database containing > 2000 environmental exposures from natural, built, and social environment domains, and found that exposure to cigarette consumption, ethyl dichloride and ethylene oxide, and PM2.5, were more closely associated with lung cancer. A literature review by Gracia-Cazaña et al.^37^ suggested that physical–chemical substances, living entities (such as polyomavirus and human papillomavirus), and lifestyle factors made up the exposome of skin cancer, and this set of environmental exposures could determine the incidence of skin cancer. One study on pancreatic cancer (PCa) used the exposome approach to extract variables from general external, internal external, and specific external domains^38^. This study identified that several exposome factors were related to PCa alone and in combination with other exposures. Resultingly, the practical application of the exposome approach has been widely used in various epidemiological fields, however, the exposome-CRC studies are lacking.

Previous studies have demonstrated that certain exposome factors (e.g. lifestyle) are related to CRC, however, the application of the exposome theoretical framework, and the extent to which the exposome domain can modulate the risks of CRC, remains unknown. Therefore, based on the model of Vermeulen et al.^20^ and following the exposome approach, our study aimed to construct valid exposome measurements and examine the relationship between exposome counts and the risk of CRC, which will inform future strategies for the prevention and early detection of CRC.

## Methodology

### Data source

The UKB is a population-based cohort study on > 500,000 people in the UK. Participants aged 40–69 years were recruited from 22 assessment centres across England, Scotland, and Wales between 2006 and 2010^44^. Participants’ socioeconomic information, lifestyle (e.g. diet, alcohol, and sleep), and other health factors were obtained via a self-completed touchscreen questionnaire and an interview at enrolment^45^.

### Measurement

#### Outcome

The CRC cases were coded using the International Classification of Diseases, 9th (ICD-9), or 10th Revision (ICD-10). The codes included ICD-9 153–1541, 2113, 2114, 2303, 2004, 1975, and 2352, or ICD-10 C180, C182–C189, C19, C20, D12–D129, D10, C785, and D374.

#### Exposome variables

Based on the conceptual framework, we initially identified 12 items related to exposome factors. These factors were physical activities, diet, drinking, smoking, obesity, low income, unemployment, high school, seldom confiding in someone, feel isolated, garden percentage (300 m buffer), natural environment percentage (300 m buffer). The definitions of the variables can be seen in Appendix Table S1.

**Figure 2:**
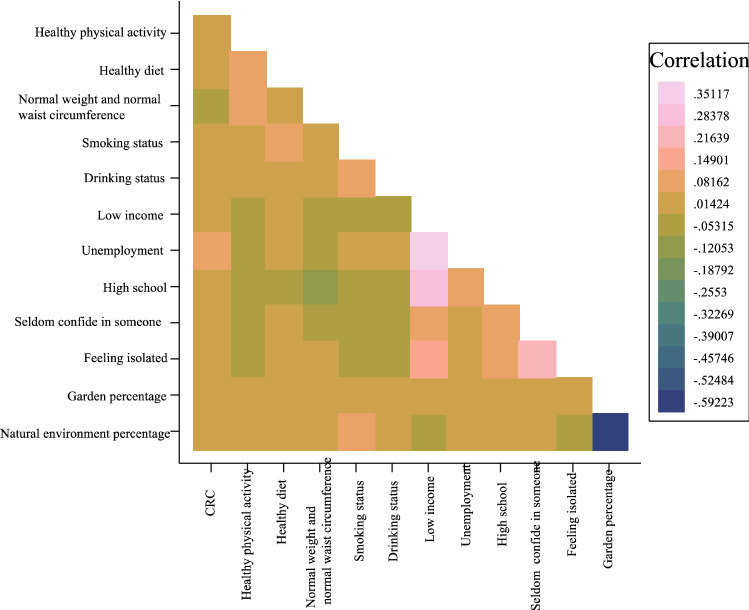
Heatmap of correlation between variables.

Healthy physical activity was coded as 1 if residents had < 150 min/week moderate, < 75 min/week vigorous, or < 150 min/week mixed (moderate + vigorous) activity. A healthy diet was coded as 1 if the respondents ate ≥ 4 ideal food groups. The ideal food groups included: fruits, ≥ 3 servings/day; vegetables, ≥ 3 servings/day; fish, ≥ 2 servings/week (counted by oily and non-oily fish); processed meat, ≤ 1 serving/week; and unprocessed meat, ≤ 1.5 serving/week. Smoking status was coded as 1 for those who had never smoked or had previously smoked. Drinking status was coded as 1 if participants drank moderately. Normal weight and waist circumference were coded as 1 if an individual’s BMI was ≤ 25 kg/m^2^ and they had a normal waist circumference (cm) (women < 88 cm; men < 102 cm) what were regarded as low-risk lifestyle factors. Low income was coded as 1 if an individual’s household income was < €30,999. Unemployment was coded as 1 if respondents were not in paid employment or self-employed. High school was coded as 1 if people had a high school degree or lower. Seldom confiding in someone was coded as 1 if people were unable to confide in someone close to them less than once a month or less than once every few months. Feeling isolated was coded as 1 if they felt isolated. In the UKB database, garden percentage (300 m buffer) was defined as the percentage of the home location buffer classed as ‘Domestic garden’ with home location data buffered at 300 m. Natural environment percentage (300 m buffer) was defined as the percentage of the home location buffer classed as ‘Natural Environment’ in the Land Cover Map (LCM) 2007 with home location data buffered at 300 m. Figure 2 shows the correlation between variables.

#### Covariates

Sociodemographic characteristics included age at baseline and sex. Family history (including parents and siblings) of cancer events, cancer screening, and Townsend deprivation index were also controlled in our study.

#### Factor analysis

We used factor analysis to determine whether these items could be combined into separate components reflecting different aspects of environmental exposure. We also performed Kaiser–Meyer–Olkin (KMO) and Butler sphericity tests, which showed that these variables were suitable for factor analysis. The factors were rotated using the varimax method to achieve a simpler structure with greater interpretability. An eigenvalue > 1.0 was detained. Finally, only exposome factors with loading ± 0.4 were regarded as important in this study^46^. Five domains were extracted from the exposome items, according to the rule that eigenvalues were > 1.0, and varimax rotation was performed to minimise loading complexity for each component.

Component 1 represented three items pertaining to lifestyle (namely healthy physical activity, healthy diet, and normal weight and waist circumference). Component 2 represented two items pertaining to less tobacco and alcohol use (namely smoking and drinking status). Component 3 represented three items pertaining to concern about social economics (namely low income, unemployment, and high school). Component 4 represented three items pertaining to social support (namely seldom confiding in someone, feel isolated). Component 5 represented two items pertaining to ecosystem (namely garden and natural environment percentage). The coefficients for the items are displayed in Table 1.

**Table 1:** Matrix of factors (absolute value).

Variables	Factor 1	Factor 2	Factor 3	Factor 4	Factor 5
Healthy physical activity	**0.5676**	0.0552	0.0135	0.1215	0.0304
Healthy diet	**0.5749**	0.1481	0.1295	0.0564	0.0095
Normal weight and normal waist circumference	**0.6366**	0.1554	0.1157	0.0060	0.0128
Smoking status	0.0005	**0.7479**	0.0017	0.0758	0.0253
Drinking status	0.0023	**0.6558**	0.0603	0.0237	0.0349
Low income	0.0123	0.1016	**0.8095**	0.1202	0.0316
Unemployment	0.0442	0.1191	**0.7654**	0.0440	0.0243
High school	0.2809	0.2127	**0.4736**	0.0107	0.0063
Seldom confiding in someone	0.0438	0.0413	0.0400	**0.7665**	0.0053
Feel isolated	0.0160	0.1028	0.0518	**0.7572**	0.0152
Garden percentage (300 m)	0.0077	0.0658	0.0068	0.0334	**0.9004**
Natural environment percentage (300 m)	0.0071	0.0670	0.0140	0.0404	**0.9016**

Significant values (loadings over 0.4) are in [bold].

### Statistical analysis

The 12 exposome items were aggregated into 5 components based on factor analysis (principal components method). First, we described the sociodemographic and exposome-related characteristics of the respondents, and compared between-group differences using one-way ANOVA or χ^2^ test when appropriate. Next, we conducted a full sample analysis to examine the relationship between all exposome variables and the risk of CRC. Thirdly, factors were employed in multivariable regression models called principal component regression analyses^46–49^. Cox proportional hazard models were used to estimate the CRC risk hazard ratios (HRs) and 95% confidence intervals (CIs) related to the exposome factors. Fourthly, we also summed the exposome counts within each domain and examined the cumulative effect of exposome domains on CRC risk. All analyses were conducted using STATA software (version 15.0; StataCorp, TX, USA), and two-tailed *p*-values ≤ 0.05 were considered statistically significant.

### Ethics approval

The UKB study was approved by the National Information Governance Board for Health and Social Care in England and Wales, and the Community Health Index Advisory Group in Scotland and the North West Multicenter Research Ethics Committee. All participants provided written informed consent. This study was approved by the Ethical Committee of Peking University (Beijing, China). In addition, all methods were performed in accordance with the relevant guidelines and regulations.

## Results

### Descriptive statistics

Acquired data from the UKB participants with outcome data was 502,528. After excluding 167,158 samples with missing values, the final sample size was 335,370, and 10,702 cases of CRC were identified. As shown in Table 2, the mean age of participants who developed CRC was 59.86. Among the CRC cases, 57.93% and 42.07% were male and female, respectively. Moreover, 11.46% of these cases reported a family history of CRC events.

**Table 2:** A statistical description of respondents’ characteristics (n = 335,370).

Characteristic	All Participants (n = 335,370)	Participants who developed colorectal cancer(n = 10,702)	Participants who did not developed colorectal cancer (n = 324,668)	*p* value
**Control variables**				
Age, years				
Mean age at baseline, mean (SD)	56.12 (8.082)	59.86 (6.72)	55.99 (8.09)	0.007
≤ 55, N (%)	147,980 (44.12%)	2539 (23.72%)	145,441 (44.80%)	0.006
55–65, N (%)	128,996 (38.46%)	5095 (47.61%)	123,901 (38.16%)	
≥ 65, N (%)	58,394 (17.41%)	3068 (28.67%)	55,326 (17.04%)	
Gender				
Female, N (%)	178,202 (53.14%)	4502 (42.07%)	173,700 (53.50%)	0.000
Male, N (%)	157,168 (46.86%)	6200 (57.93%)	150,968 (46.50%)	
Family History of CRC events				
Yes, N (%)	22,647 (6.75%)	1226 (11.46%)	21,421(6.60%)	0.000
No, N (%)	312,723 (93.25%)	9476 (88.54%)	303,247 (93.40%)	
Screening				
Yes, N (%)	104,759 (31.24%)	4608 (43.06%)	100,151 (30.85%)	0.000
No, N (%)	230,611 (68.76%)	6094 (56.94%)	224,517 (69.15%)	
Deprivation				
Mean (SD)	0.274 (0.446)	0.282 (0.45)	0.273 (0.446)	0.000
**Exposome variables**				
Healthy diet				
Yes, N (%)	43,366 (12.93%)	1284 (12.00%)	42,082 (12.96%)	0.003
No, N (%)	292,004 (87.07%)	9418 (88.00%)	282,586 (87.04%)	
Healthy physical activity				
Yes, N (%)	240,680 (71.77%)	7298 (68.19%)	233,382 (71.88%)	0.000
No, N (%)	94,690 (28.23%)	3404 (31.81%)	91,286 (28.12%)	
Smoking status				
Yes, N (%)	301,569 (89.92%)	9461 (88.40%)	292,108 (89.97%)	0.000
No, N (%)	33,801 (10.08%)	1241 (11.60%)	32,560 (10.03%)	
Drinking status				
Yes, N (%)	169,255 (50.47%)	4977(46.51%)	164,278 (50.60%)	0.000
No, N (%)	166,115 (49.53%)	5725 (53.49%)	160,390 (49.40%)	
Normal weight and normal waist circumference				
Yes, N (%)	93,945 (28.01%)	2227 (20.81%)	91,718 (28.25%)	0.000
No, N (%)	241,425 (71.99%)	8475 (79.19%)	232,950 (71.75%)	
Low income				
Yes, N (%)	157,019 (47.82%)	6134 (57.32%)	150,885 (46.47%)	0.000
No, N (%)	178,351 (53.18%)	4568 (42.68%)	173,783 (55.53%)	
Unemployment				
Yes, N (%)	128,909 (38.44%)	5587 (52.21%)	123,322 (37.98%)	0.000
No, N (%)	206,461 (61.56%)	5115 (47.79%)	201,346 (62.02%)	
High school				
Yes, N (%)	199,792 (59.57%)	6912 (64.59%)	192,880 (59.41%)	0.000
No, N (%)	135,578 (40.43%)	3790 (35.41%)	133,788 (40.59%)	
Seldom confiding in someone				
Yes, N (%)	84,217 (25.11%)	2886 (26.97%)	81,331 (25.05%)	0.000
No, N (%)	251,153 (74.89%)	7816 (73.03%)	243,337 (74.95%)	
Feel isolated				
Yes, N (%)	60,274 (17.97%)	1971 (18.42%)	58,303 (17.96%)	0.223
No, N (%)	275,096 (82.03%)	8731 (81.58%)	266,365 (82.04%)	
Garden percentage (300 m)				
Mean (SD)	31.083 (14.79)	30.374 (14.38)	31.11 (14,81)	0.000
Natural environment percentage (300 m)				
Mean (SD)	26.94 (25.62)	25.143 (25.14)	26.92 (25.64)	0.000

#### Full sample analysis

**Figure 3:**
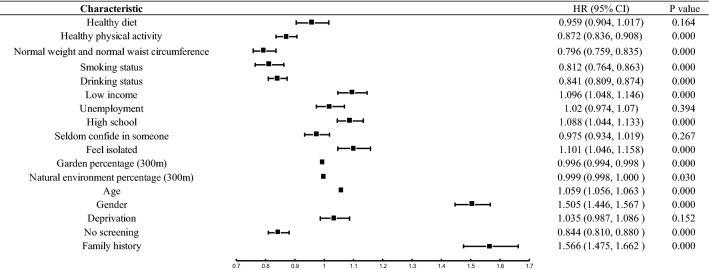
Effects of exposome variables on the risk of CRC in the UKB cohort.

Figure 3 shows the effects of all exposome variables on the risk of CRC in the UKB cohort. According to Fig. 3, participants who exercised regularly, had normal weight and normal waist circumference, never have smoked, had low to moderate drinking, and lived in areas with more garden coverage and natural environment coverage were related to the reduced risk of CRC. Those who were low-income, unemployed, less educated, and isolated were significantly associated with an increased risk of CRC.

Table 3 shows the multivariable-adjusted HRs (95% CI) of CRC events by exposome factors among the 335,370 participants. The results from the Cox models showed that a healthy lifestyle (HR = 0. 889, 95% CI 0.871–0.907; *p* < 0.001), and less tobacco and alcohol use (HR = 0.892, 95% CI 0.876–0.909; *p* < 0.001) were positively associated with a reduced risk of CRC. Adverse results were identified among the socioeconomic and social support domains. Disadvantageous socioeconomic status (HR = 1.081, 95% CI 1.058–1.105; *p* < 0.001) and insufficient social support (HR = 1.036, 95% CI 1.107–1.056; *p* < 0.001) were associated with an increased risk of CRC. The ecosystem was positively correlated with a reduced risk of CRC (HR = 0.97, 95% CI 0.952–0.989; *p* < 0.01).

**Table 3:** Multivariable-adjusted hazard ratio (95% CI) of CRC events by exposome factors among 335,370 participants.

Variables	Hazzard ratio (95% CI)	*p* value^a^
Factor 1 Lifestyle	0.889*** (0.871, 0.907)	0.000
Factor 2 Less tobacco and alcohol use	0.892*** (0.876, 0.909)	0.000
Factor 3 Social economic	1.081*** (1.058, 1.105)	0.000
Factor 4 Social support	1.036*** (1.017, 1.056)	0.000
Factor 5 Ecosystem	0.970** (0.952, 0.989)	0.002
Age	1.059*** (1.055, 1.062)	0.000
Gender	1.492*** (1.435, 1.552)	0.000
Deprivation	1.078*** (1.032, 1.125)	0.001
No screening	0.844*** (0.810, 0.879)	0.000
Family history of CRC events	1.565*** (1.474, 1.661)	0.000

^a^*p* < 0.05*; *p* < 0.01**;*p* < 0.001***.

### Exposome counts and CRC

Based on a study by Safford et al.^50^, we decided to use exposome factor counts to examine the cumulative effects of different exposome domains on CRC events. We used lifestyle, social, and ecosystem domains to construct the exposome counts. Notably, exposome counts were categorised into three parts: lifestyle, social, and ecosystem counts. Lifestyle counts were developed by combining physical activities, diet, drinking, smoking, and obesity. The counts range was 0–5. A higher count indicated a healthier lifestyle. Social counts were developed by combining low income, unemployment, high school, seldom confiding in someone, feel isolated. The counts range was 0–5, where a higher count indicated that participants were more socially disadvantaged. Ecosystem counts were developed by combining the median garden percentage (300 m; above the median coded as 1) and the median natural environment percentage (300 m; above the median coded as 1). This count range was 0–2, with a higher score indicating a better ecosystem. Table S2 shows the exposome counts of the respondents.

**Figure 4:**
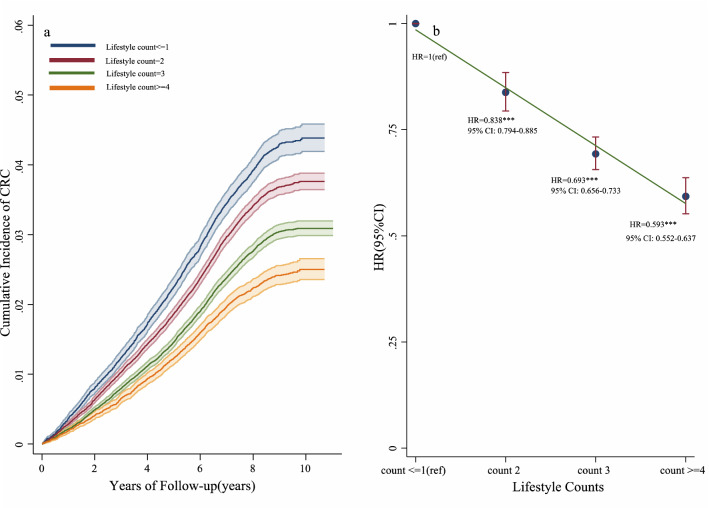
Effects of healthy lifestyle counts on the risk of incident CRC in the UKB cohort.

We divided participants into four lifestyle dimension categories: ≤ 1count group (48,790 of 335,370 participants [14.55%]), 2 counts group (114,450 participants [34.13%]), 3 counts group (118,456 participants [35.32%]), and ≥ 4 counts group (53,674 participants [16.00%]). As shown in Fig. 4a, the cumulative incidence of CRC decreased with increasing lifestyle counts. Compared with the ≤ 1 lifestyle count, the relative risk of CRC incidence was lower in participants with ≥ 4 lifestyle counts (HR = 0.593, 95% CI 0.552–0.637; *p* < 0.001; Fig. 4b).

**Figure 5:**
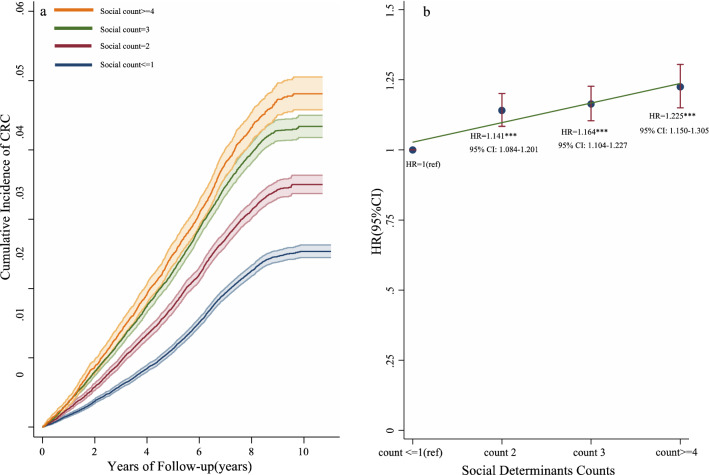
Effects of disadvantageous social determinants counts on the risk of incident CRC in the UKB cohort.

The social dimension was categorised into four groups: ≤ 1 count group (140,587 of 335,370 participants [41.92%]), 2 counts group (85,737 participants [25.56%]), 3 counts (72,728 participants [21.69%]), and ≥ 4 counts group (36,318 participants [10.83%]). We observed an increased cumulative incidence of CRC with increasing social counts (Fig. 5a). The relative risk of CRC incidence was higher in participants with ≥ 4 social domain counts (HR = 1.225, 95% CI 1.150–1.305; *p* < 0.001) than in those with a social domain count of ≤ 1 (Fig. 5b).

**Figure 6:**
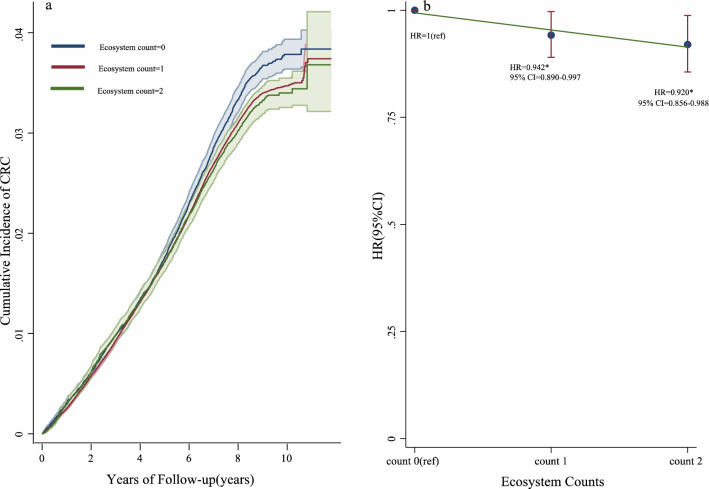
Effects of ecosystem counts on the risk of incident CRC in the UKB cohort.

We divided participants into three ecosystem dimension participants: 0 count group (49,158 of 335,370 participants [14.66%]), 1 count group (233,209 participants [69.54%]), and 2 counts group (53,003 participants [15.80%]). A decreased cumulative incidence of CRC was observed when ecosystem counts were added (Fig. 6a). The relative risk of CRC incidence was lower in participants in the 2 counts group (HR = 0.920, 95% CI 0.856–0.988; *p* < 0.001) than in those in the 0 count group (Fig. 6b). In addition, we also conducted an additional analysis to examine the relationship between each exposome dimension and the risk of CRC incidence independently, and also found robust results (see Table S3).

## Discussion

Numerous studies have focused on the effects of certain exposure factors on CRC events, however, few of these studies have applied the exposome theoretical framework to explore the synergy between these factors in relation to CRC development. To bridge this knowledge gap, this study used UKB data to explore the relationship between exposome factors and the risk of CRC by using evaluation measures with strong construct validity. This study has three noteworthy findings, based on the exposome theoretical framework. These results suggest that lifestyle, social, and ecosystem domains are related to the risk of CRC.

First, in terms of lifestyle domain, we found that a healthy lifestyle and less tobacco and alcohol use were associated with a reduced risk of CRC. These results are in agreement with those of previous studies^10–13^. A healthy diet is considered to contain rich fish, fruits, vegetables, and less processed and red meat^12^. This diet pattern included sufficient nutrition, and less fatty and carcinogenic substances, which could reduce the risk of CRC^51^. Obesity has a direct effect on certain hormone levels, such as insulin, oestrogen, and insulin-like growth factor-1, which produce a favourable environment for carcinogenesis^52^. Physical activity can reduce CRC risk by motivating gut motility, benefiting the immune system, and elevating metabolic efficiency^51,53^. Alcohol metabolites, such as acetaldehyde, increased the risk of CRC, because acetaldehyde was evaluated as a carcinogen^54^. Smoking induces angiogenesis and suppresses cell-mediated immunity to facilitate tumour growth^55^.

Second, in terms of social domain, this study suggested that lower SES and less social support were associated with an increased risk of CRC. The finding of a relationship between SES and CRC risk was consistent with that of previous studies. Higher incidences of CRC are related to greater social disadvantage^26,56^. One possible reason is that low-SES people may have less rational health behaviour, know less about their symptoms, and communicate more poorly than high-SES people with health care staff^57^. In contrast, high-SES people have better access to health information^57^ and seek health services^58^, they are more likely to have behavioural changes to a healthy lifestyle^59^ and thus reduce the risk of CRC. However, findings regarding the association between social support and the incidence of CRC have been inconsistent. One study found no significant relationships between social networks and CRC incidence^28^, while Ikeda et al. found that lower social support was associated with a higher incidence of CRC among men^29^. This study supports the finding that lower support was related to an increased risk of CRC. One possibility is that social support raises the esteem of individuals and makes them feel valued, therefore, they may take better care of themselves and be more receptive to preventative services^29,60^. Additionally, social support reduces stress and depression. Fewer stress hormones reduce the risk of immune dysregulation caused by^61^, thereby suppressing the environment for tumour initiation.

Third, in terms of the ecosystem domain, a better ecosystem was associated with a reduced risk of CRC. However, limited studies have evaluated the ecosystem and CRC incidence. Increasing studies have suggested that ecosystems play a role in the incidence of cancer^30,31^. It is unclear how ecosystems influence the risk of CRC. One possibility was that a better ecosystem was related to a more covered natural environment, with less pollution and radiation^62^. Pollutants in water^35^, poisonous heavy metals^18^ and radiation are associated with an increased risk of CRC. Furthermore, a better ecosystem may provide a greener environment, which provides a venue for physical activity^63^. Engaging in more physical activity can help reduce the risk of CRC^54^.

This study has several strengths. First, the results highlight the effects of the physical (ecosystem) and social environments (SES and social support) on CRC events. The majority of previous studies have focused on the influence of biological factors on CRC events while ignoring environmental effects. Second, because of the increased acceptance that non-genetic factors play a role in diseases, this study included the entire set of exposome factors from a holistic perspective. It also showed the whole mechanism of exposome factors on the risk of CRC at a macro level. Under the exposome framework, a broad approach for CRC prevention can be considered. Apart from promoting a healthy lifestyle among the population to prevent CRC, it is also important to take care of people with lower SES and less social support.

However, this study has several limitations. First, this study did not capture temporal changes in exposome factor changes (e.g. lifestyle and SES changes) due to the nature of the dataset. Future studies should use panel data to trace the impact of dynamic changes in exposome factors on CRC events. Second, due to the collinearity of variables in the physical–chemical domain, we did not conduct a full exposome analysis. Future studies will reduce the collinearity and add physical–chemical factors into the full exposome analysis. Third, there may be complex interactions between the three domains, which were not observed in detail in this study. Future studies should include mediation or pathway analysis to explore the interaction of lifestyle, social, ecosystem effects on the risk of CRC. Fourth, although our analyses controlled for various confounding factors, we may have overlooked other factors related to CRC due to the limitation of using secondary datasets. Future studies should use mixed methods (combing semi-structured interviews, and a case study with quantitative methods) to improve the robustness of the results.

## Conclusion

To the best of our knowledge, this is the first study to focus on the exposome framework and CRC in social epidemiology studies. Our study included the whole set of exposome factors from a holistic perspective and employed factor analysis to improve the understanding of the relationship between exposome domains and CRC events. These findings suggest that lifestyle, social, and ecosystem domains are related to CRC events. Similar risk trends were also observed across the exposome count group with CRC incidence. This study confirmed the relationship between exposome factors and CRC events from an empirical perspective, which would provide policy implications for future CRC prevention.

## Supplementary Information

Supplementary Information.

## Data Availability

Data are available in a public, open access repository. This research has been conducted using the UK Biobank Resource under Application Number 44430. The UK Biobank data are available on application to the UK Biobank (https://www.ukbiobank.ac.uk/).
