# Supplementary material for: Exposome approach for identifying modifiable factors for the prevention of colorectal cancer
Source: Sci Rep. 2022 Dec 14;12:21615. doi: 10.1038/s41598-022-25832-9 (PMC9750985; doi:10.1038/s41598-022-25832-9)
Supplement: Supplementary file 1 — Supplementary Information. [file 41598_2022_25832_MOESM1_ESM.docx]

**Table S1.** Definition and code of all variables

| Variables | Definition | Code |
| --- | --- | --- |
| Age | The age of the participant on the day they attended an Initial Assessment Centre. | Continuous variables |
| Gender | The gender of the respondents. | 1=male; 0=female |
| Deprivation | Townsend deprivation index. | Continuous variables |
| No screening | Respondents never had a screening test for bowel (colorectal) cancer? | 1=yes; 0=no |
| Family history of CRC events | Father, mother or siblings ever diagnosed colorectal cancer. | 1=yes;0=no |
| Healthy diet | Respondents eat ≥4 ideal food groups, ideal food groups were including fruits: ≥3 servings/day; vegetables: ≥3 servings/day; fish: ≥2 servings/week (counted by oily fish and non-oily fish); processed meat: ≤1 serving/week, unprocessed meat; ≤1.5 serving/week (counted by beef, lamb mutton, pork). | 1=yes;0=no |
| Healthy physical activity | Physical activity ≥150 min/week moderate or ≥75 min/week vigorous or 150 min/week mixed (moderate + vigorous) physical activity. | 1=yes; 0=no |
| Smoking status | Never or previous smoking. | 1=yes; 0=no |
| Drinking status | Moderate drinking: 0<women≤14g/day; 0<men≤28g/day. | 1=yes; 0=no |
| Normal weight and normal waist circumference | BMI(Body mass index) ≤25 kg/m2 and waist circumference (cm) : women < 88cm; men <102cm. | 1=yes; 0=no |
| Low income | Household income < 30,999. | 1=yes; 0=no |
| Unemployment | Not in paid employment or self-employed. | 1=yes; 0=no |
| High school | High school degree or below. | 1=yes; 0=no |
| Seldom confiding in someone | Unable to confide in someone close to less than once a month or less than once every few months. | 1=yes; 0=no |
| Feel isolated | Feel isolated. | 1=yes; 0=no |
| Garden percentage (300m) | The percentage of the home location buffer classed as 'Domestic garden', as a proportion of all land-use types, and with home location data buffered at 300m. | Continuous variables |
| Natural environment percentage (300m) | The percentage of the home location buffer classed as 'Natural Environment' in the Land Cover Map (LCM) 2007, and with home location data buffered at 300m. | Continuous variables |
| Lifestyle counts | Lifestyle counts are developed by combing, physical activities, diet, drinking, smoking and obesity. | Range from 0 to 5. A higher count indicates a healthier lifestyle. |
| Social determinants counts | Social determinants counts are developed by combing low income, unemployment, high school, less confiding in someone, feel isolated. | Range from 0 to 5, a higher score means in a lower social disadvantaged group. |
| Ecosystem counts | Ecosystem counts are developed by combing the median of garden percentage (1000m) (above the median coded as 1) and the median of natural environment percentage (1000m) (above the median coded as 1). | Range from 0 to 2. A higher score indicated a better ecosystem. |

**Table S2.** Exposome counts among 335,370 participants

| **Dimension** | **Count** | **All Participants (n=****335,370)** | **Participants who developed colorectal cancer(n=10,702)** | **Participants who did not developed colorectal cancer (n=324,668)** |
| --- | --- | --- | --- | --- |
| **Lifestyle dimension** | | | | |
|  | ≤1count group | 48,790 (14.55%) | 1,993 (18.62%) | 46,797 (14.41%) |
|  | 2 counts group | 114,450 (34.13%) | 4,044 (37.79%) | 110,406 (34.01%) |
|  | 3 counts group | 118,456 (35.32%) | 3,422 (31.98%) | 115,034 (35.43%) |
|  | ≥ 4 counts group | 53,674 (16.00%) | 1,243 (11.61%) | 52,431 (16.15%) |
| **Social dimension** | | | | |
|  | ≤1 count group | 140,587 (41.92%) | 3,327 (31.09%) | 137,260 (42.28%) |
|  | 2 counts group | 85,737 (25.56%) | 2,804 (26.20%) | 82,933 (25.54%) |
|  | 3 counts | 72,728 (21.69%) | 2,958 (27.64%) | 69,770 (21.49%) |
|  | ≥ 4 counts group | 36,318 (10.83%) | 1,613 (15.07%) | 34,705 (10.69%) |
| **Ecosystem dimension** | | | | |
|  | 0 count group | 49,158 (14.66%) | 1,633 (15.26%) | 47,525 (14.64%) |
|  | 1 count group | 233,209 (69.54%) | 7,400 (69.15%) | 225,809 (69.55%) |
|  | 2 counts group | 53,003 (15.80%) | 1,669 (15.60%) | 51,334 (15.81%) |

**Table S3.** Multivariable-adjusted hazard ratio (95% CI) of CRC events by separate exposome dimensions among 335,370 participants

| **Variables** | **Model 1:**  **Null model** | **Model 2:**  **Lifestyle dimension** | **Model 3:**  **Social dimension** | **Model 4**  **Ecosystem dimension** |
| --- | --- | --- | --- | --- |
|  | Hazzard Ratio  (95% CI) | Hazzard Ratio  (95% CI) | Hazzard Ratio  (95% CI) | Hazzard Ratio  (95% CI) |
| **Lifestyle dimension** | |  |  |  |
| ≤1count group |  | 1 (ref) |  |  |
| 2 counts group |  | 0.829^***^  (0.786, 0.875) |  |  |
| 3 counts group |  | 0.681^***^  (0.644, 0.720) |  |  |
| ≥ 4 counts group |  | 0.578^***^  (0.538, 0.620) |  |  |
| **Social dimension** | |  |  |  |
| ≤1 count group |  |  | 1 (ref) |  |
| 2 counts group |  |  | 1.161^***^  (1.103, 1.222) |  |
| 3 counts |  |  | 1.206^***^  (1.144, 1.272) |  |
| ≥ 4 counts group |  |  | 1.300^***^  (1.220, 1.384) |  |
| **Ecosystem dimension** | |  |  |  |
| 0 count group |  |  |  | 1 (ref) |
| 1 count group |  |  |  | 0.931^*^  (0.879, 0.985) |
| 2 counts group |  |  |  | 0.910^*^  (0.847, 0.978) |
| Age | 1.063^***^  (1.059, 1.066) | 1.062^***^  (1.059, 1.066) | 1.057^***^  (1.054, 1.061) | 1.063^***^  (1.060, 1.066) |
| Gender | 1.499^***^  (1.442, 1.558) | 1.477^***^  (1.421, 1.535) | 1.521^***^  (1.464, 1.581) | 1.499^***^  (1.442, 1.558) |
| Deprivation | 1.142^***^  (1.095, 1.192) | 1.104^***^  (1.058, 1.152) | 1.115^***^  (1.068, 1.163) | 1.117^***^  (1.068, 1.169) |
| No screening | 0.858^***^  (0.823, 0.894) | 0.847^***^  (0.813, 0.883) | 0.857^***^  (0.822, 0.893) | 0.858^***^  (0.823, 0.894) |
| Family history | 1.571^***^  (1.480, 1.668) | 1.566^***^  (1.475, 1.662) | 1.572^***^  (1.481, 1.669) | 1.572^***^  (1.480, 1.668) |

A: p<0.05 ^*^; p<0.01 ^**^; p<0.001^***^
